# Nutritional Profile, Antioxidative and Antihyperglycemic Properties of *Padina tetrastromatica* from Tioman Island, Malaysia

**DOI:** 10.3390/foods10081932

**Published:** 2021-08-20

**Authors:** Kishneth Palaniveloo, Liaw Yee-Yinn, Leong Jia-Qi, Alvin Chelliah, Song Sze-Looi, Thilahgavani Nagappan, Shariza Abdul Razak, Kamal Dua, Jestin Chellian, Dinesh Kumar Chellappan, Anil Philip Kunnath

**Affiliations:** 1 Institute of Ocean and Earth Sciences, Universiti Malaya, Kuala Lumpur 50603, Malaysia; 2 Department of Life Sciences, International Medical University, Bukit Jalil, Kuala Lumpur 57000, Malaysia; liawyeeyinn@gmail.com (L.Y.-Y.); ivyleong1996@yahoo.com (L.J.-Q.); jestin_chellian@imu.edu.my (J.C.); dinesh_kumar@imu.edu.my (D.K.C.); 3 Reef Check Malaysia, Suite 5.19–5.22, Wisma Central, Jalan Ampang, Kuala Lumpur 50450, Malaysia; alvinchelliah@gmail.com; 4 Institute for Advanced Studies, Universiti Malaya, Kuala Lumpur 50603, Malaysia; szelooi@um.edu.my; 5 School of Marine and Environmental Sciences, Universiti Malaysia Terengganu, Kuala Terengganu 21030, Malaysia; 6 Institute of Marine Biotechnology, Universiti Malaysia Terengganu, Kuala Terengganu 21030, Malaysia; 7 Nutrition and Dietetics Program, School of Health Sciences, Health Campus, Universiti Sains Malaysia, Kubang Kerian 16150, Malaysia; 8 Discipline of Pharmacy, Graduate School of Health, University of Technology Sydney, Ultimo, NSW 2007, Australia; Kamal.Dua@uts.edu.au; 9 Australian Research Centre in Complementary and Integrative Medicine, Faculty of Health, University of Technology Sydney, Ultimo, NSW 2007, Australia; 10 Division of Applied Biomedical Science and Biotechnology, School of Health Sciences, International Medical University, Bukit Jalil, Kuala Lumpur 57000, Malaysia

**Keywords:** seaweed, brown algae, *Padina tetrastromatica*, Tioman Island, functional food, nutrition, antioxidant, antidiabetic, antihyperglycemic

## Abstract

Seaweeds are an important ingredient of functional foods recommended for daily food, due to their unique compositions and nutritional value. *Padina tetrastromatica* is a brown edible seaweed that is commonly found along the coastal regions of Peninsular Malaysia and consumed as food by some coastal communities. This study investigates the nutritional and antihyperglycaemic potential of *P. tetrastromatica* extracts, which is generally accepted as an important functional food. In our methodology, we induced diabetes intraperitoneally in experimental animals with a dose of 65 mg kg^−1^ body weight of streptozotocin. Oral treatment with 200 and 400 mg kg^−1^ of *P. tetrastromatica* ethanolic and ethyl acetate extracts were initiated, respectively, to experimental rats once daily for 18 days. Metformin was used as the positive control. Biochemical estimations and histopathological analysis were included in this study. Treatment with *P. tetrastromatica* extracts significantly lowered the plasma glucose levels in Streptozotocin-induced diabetic rats. In addition, *P. tetrastromatica* extract treatment also showed a significant reduction in serum alanine transaminase levels. However, no significant changes were observed in serum aspartate transaminase levels. The ethyl acetate extract of *P. tetrastromatica* at 400 mg kg^−1^ dose shows some nephroprotective effect, which is observed from the significant increase in the plasma albumin levels. Histopathological evaluation revealed no marked morphological changes in tissues of the isolated organs of the ethyl acetate extract-treated group, revealing the safe nature of *P. tetrastromatica*.

## 1. Introduction

Marine macroalgae are one of nature’s most biologically active resources that has been used as both medicinal and food ingredients traditionally by communities globally [1]. Approximately 250 macroalgal species is reported to have commercial value, where at least 150 species are consumed as food, supplement or local delicacies. They are a vital source of nutrition; rich in dietary fibre, low lipid content, high polysaccharide concentration, rich in minerals, polyunsaturated fatty acids and vitamins [2]. This makes macroalgae vital for lowering blood cholesterol and glucose, capable of increasing faecal bulk, decreasing intestinal transit time, lowering the occurrence of diabetes, obesity, heart diseases and even cancer [3]. Marine macroalgae are known to be a rich source of pharmacologically active compounds, such as antibiotic, antiviral and anticancer activities [4].

A recent survey by Knorr and WWF (2019) which evaluated nutritional properties and environmental impact, flavour, accessibility, acceptability and affordability, ranks seaweed highly in a list of fifty edible resources. The nutritional and pharmacological potential of marine macroalgae drives our continuous interest in investigating this edible seaweed. Our previous investigations evaluated the nutritional and bioactivity of wild and cultured *Gracilaria manilaensis* [5] and *Caulerpa racemosa* [6]. Recently, we came across a population of *Padina tetrastromatica* at Kampung (village) Tekek at Tioman Island of Eastern Peninsular Malaysia. *P. tetrastromatica* (Class, Phaeophyceae; Family, Dictyotaceae) gets washed up on the shore of Kampung Tekek during monsoon, causing an unpleasant sight for the tourism activity at the island. As an important functional food, *P. tetrastromatica* is a known source of glucuronic, uronic acid, alginic acid, sulphated polysaccharides (SPSs) and is reported to exhibit antihyperglycaemic potential [7].

According to the World Health Organisation (WHO), an estimated 2.2 million deaths attributable to high blood glucose in 2012 constituted 50% of people aged below 70 years old. WHO projected diabetes to be the seventh leading cause of death in 2030 [8]. Therapeutic options available for diabetes are oral hypoglycaemic agents (OHA), insulin therapy and lifestyle modifications. However, side effects, such as hypoglycaemia or treatment failures in patients, calls for alternative natural source of treatment [9]. Keeping that in mind, we characterised the nutritional properties of *P. tetrastromatica* and its antihyperglycaemic potential in Streptozotocin (STZ)-induced rats.

## 2. Materials and Methods

### 2.1. Collection, Identification and Processing of Algal Material

*Padina tetrastromatica* was collected from five different locations along the beach of Kampung Tekek at Tioman Island, Pahang, Malaysia (*n* = 3). Approximately 5 kg wet weight (kg *w*/*w*) each, was hand-picked and treated separately. Sample identification was confirmed based on morphology for genus and molecular technique for species. Approximately 2 g of fresh sample was kept for molecular identification. Herbarium voucher (UMTP1901) was deposited at the Faculty of Marine and Environmental Sciences herbarium of Universiti Malaysia Terengganu. The seaweed samples were cleaned in freshwater to remove sediment and then deep-frozen at −20 °C for 48 h prior to freeze-drying (ModulyoD, Thermo Electron Corporation, Waltham, MA, USA) for another 48 h. Freeze-dried algae samples were ground in a mechanical grinder (IKA, A 11 Basic, Berlin, Germany), to obtain homogeneous powder (particles ≤ 500 µm), and kept at room temperature over silica gel until further use.

### 2.2. Molecular Identification of the Sample

Samples were rinsed with seawater, followed by distilled water and ultrapure quality water. Total DNA from the *P. tetrastromatica* sample was extracted using the i-genomic Plant DNA Extraction Mini Kit (iNtRON Biotechnology Inc., Gyeonggi, Korea) following the manufacturer’s instructions. Polymerase chain reaction (PCR) was carried out using primer-pairs trnY-P1 (5′-TCYATCRTAGGTTCGAATCC-3′) and cox3-P6.3 (5′-CCWACDATHGCRTGATGVGCCC-3′) [10]. The specimen was confirmed to be *P. tetrastromatica* as determined by BLAST search against GenBank database with 98% identity.

### 2.3. Proximate Analysis

Moisture content was determined gravimetrically by quantifying the weight loss of the sample (3 g, *n* = 3) by drying (Memmert UFP 600, Buechenbach, Germany) at 105 °C according to AOAC 934.01 protocol. Ash content was quantified after incineration in a muffle furnace (Barnstead Thermolyne, Ramsey, MN, USA) at 550 °C [5]. Total protein content of samples was calculated according to the Kjeldahl method (*n* = 3) using a Foss Kjeltec system (FOSS, Hilleroed, Denmark) [5]. The total lipids were obtained by soxhlet extraction with chloroform: methanol 2:1 (*v*/*v*) for 4 h. Carbohydrate contents were calculated using the formula:

Carbohydrates = [100% − (% protein + % lipid + % ash + % moisture)]

The gross calorific content was estimated using the Isoperibol oxygen bomb calorimeter (IKA Calorimeter System C 2000 basic, Staufen, Germany) standardised with benzoic acids as described by Aroyehun et al. (2019) [5]. The results (*n* = 3) were expressed on a dry weight (DW) basis.

### 2.4. Fatty Acid Determination

Chromatography on silica gel with a mobile phase of Hexane (Hex): Ethyl acetate (EtOAc) (9:1) was used to isolate the fatty acids from the crude lipid extract of *P. tetrastromatica*. The fatty acid (FA) isolate was converted to methyl esters by transmethylation using sodium methoxide solution. 100 mg of concentrated fatty acid extract (*n* = 3) was added with 2.7 mL of Hex and 0.3 mL of 2 M sodium methoxide solution and constantly mixed at room temperature for 3 h. The resulting yellowish oil was subjected to profiling using a Shimadzu QP-2010 gas chromatograph (GC) equipped with a silica BPX70 capillary column (60 m, with a film thickness of 0.25 µm) according to Nagappan and Vairappan (2014) Identification and quantification of fatty acid methyl esters (FAMEs) was compared to those of pure FAME standards (Sigma-Aldrich, St. Louis, MO, USA). The concentration of FAME was calculated and expressed as a percentage of FAs in the lipid fraction [11].

### 2.5. Amino Acid Analysis

100 mg of powdered *P. tetrastromatica* (*n* = 3) was hydrolysed with 1 mL of 6 N hydrochloric acid (HCl) at 100 °C, followed by the addition of 1 mL 0.1 N HCl: Ethanol (EtOH) mixture (1:1, *v*/*v*) in chilled condition. Aliquots of hydrolysates (25 µL) were mixed with 10 N sodium hydroxide in a 1:1 ratio, and 1 mL of derivatisation reagent (o-phthalaldehyde (OPA) and 9-fluorenylmethyl chloroformate (FMOC) (Sigma-Aldrich (Steinheim, Germany)). The amino acid content was determined based on AOAC 999.13 using a High-Performance Liquid Chromatography (HPLC) at 335 nm ultraviolet range using a C18 column (i.d., 4.6 × 180 mm, Agilent Technologies, Santa Clara, CA, USA) at 40 °C according to Aroyehun et al. (2019). The concentration of amino acids was calculated and expressed as a percentage [5].

### 2.6. Mineral and Heavy Metal Analysis

500 mg freeze-dried *P. tetrastromatica* was subjected to wet hydrolysis in a high-pressure polytetrafluoroethylene vessel, containing 6 mL of 65% HNO_3_ and 2 mL of 35% H_2_O_2_ and digested in an Anton Paar microwave. After digestion, filtered samples are diluted to a final volume of 50 mL and analysed in an Agilent 7700 series ICP-MS (Agilent Technologies, Inc., Santa Clara, CA, USA) for multi-mineral elements based on the protocol of Aroyehun et al. (2020) [6].

### 2.7. Nonnutritive Components and Biological Activities

#### 2.7.1. Total Phenolic Content (TPC) and Flavonoid Content (TFC)

Total phenolic content was determined by using a Folin–Ciocalteau (FC) assay as described by Aroyehun et al. (2020). A calibration curve of gallic acid (25–200 µg mL^−1^) was prepared (R^2^ = 0.999). The percentage of total phenolics was calculated and expressed as milligram gallic acid equivalent (mg GAE) g^−1^ dried plant material. Total flavonoid content was determined also based on the protocol described by Aroyehun et al. (2020). A calibration curve of quercetin (25–250 µg mL^−1^) was prepared (R^2^ = 0.997), and percentage of total flavonoids was reported as milligram quercetin equivalent per gram extract (mg QE g^−1^ dried material. All samples were prepared in triplicates (*n* = 3) [6].

#### 2.7.2. Total Antioxidant Activity (TAA)—Phosphomolybdate Assay

The total antioxidant activity was performed based on the protocol by Aroyehun et al. (2020). The standard curve of gallic acid was linear between 50 and 250 µg mL^−1^ (R^2^ = 0.994). The TAC was expressed as milligram gallic acid equivalent per gram of extract (mg GAE g^−1^) [6].

#### 2.7.3. Reducing Power Capacity

The reducing power capacity was based on the protocol by Aroyehun et al. (2020). The standard curve of ascorbic acid was linear between 50 and 250 µg mL^−1^ (R^2^ = 0.994). The reducing power capacity was expressed as milligram ascorbic acid equivalent per gram of extract (mg AAE g^−1^) [6].

#### 2.7.4. Hydrogen Peroxide (H_2_O_2_) Scavenging Assay

The Hydrogen Peroxide (H_2_O_2_) scavenging assay was adapted from the protocol of Aroyehun et al. (2020). The standard curve of AA was linear between 50 and 250 µg mL^−1^ (R^2^ = 0.996) [6]. The abilities to scavenge the H_2_O_2_ were calculated as the following equation and reported in percentage activity:

H_2_O_2_ scavenging activity = (1 − absorbance of sample/absorbance of the sample) × 100

### 2.8. In Vivo Antidiabetic Study

*Padina tetrastromatica* separately extracted in 80% EtOH and EtOAc were screened for their possible in vivo antihyperglycemic potential and oral glucose tolerance efficiency (OGTT). In addition, body weight analysis, daily food intake and biochemical parameters like glycated haemoglobin, albumin, serum aspartate transaminase (AST) and alanine transaminase (ALT) were determined. At the end of the study period, the animals were sacrificed, and key organ samples were collected for histopathological analysis.

#### 2.8.1. Experimental Animals

Forty-two (42) 6-week-old Sprague Dawley (SD) male rats with an average body weight of 95 g were purchased from the Institute for Medical Research Malaysia to carry out the in vivo studies. The initial weights of the rats used for this experiment were rang-ing from 86.5 to 103.5 g. All the studies involving experimental animals were ethically conducted after obtaining the necessary approval from the local Animal Care and Use Committee (ACUC) of the International Medical University Malaysia (Project approval ID BP1-01/2018(45)). The animals were housed in clean metabolic cages in a well-ventilated house with the following conditions; temperature: 23 ± 1 °C, photoperiod: 12 h natural light and 12 h dark, humidity: 45–50% with free access to 200 commercial pelleted rat chow and water for 7 days prior to experiment [6]. The rats were divided into seven (7) groups of six (6) rats, as shown in Table 1.

Group I served as the control group in this study. In Groups II to VII, diabetes was induced through an intraperitoneal injection of 65 mg kg^−1^ Streptozotocin (STZ) (SIGMA Chemicals Co., St. Louis, MO, USA). After 72 h, the rats with blood glucose levels exceeding 11.1 mmol L^−1^ on an On Call^®^ Vivid Glucometer were included in this study. Treatment was administered in low (200 mg kg^−1^) and high (400 mg kg^−1^) doses of extracts. TWEEN 80 suspended EtOH extract was administered to Group III and Group IV, respectively, while EtOAc extract was given to Group V and Group VI in the order of high and low dose, respectively. Group VII was administered with 180 mg kg^−1^ of metformin as a positive control. All treatment doses were administered orally through a feeding tube once daily for 18 days. Animals received a standard pellet diet and purified water for 24 h over a period of 18 days. The food intake and body weights were then measured daily for 18 days.

#### 2.8.2. Biochemical and Histological Studies

At the end of the 18-day treatment period, all rats were anaesthetised using diethyl ether for dissection. Blood samples were collected using the retro-orbital sinus method in VACUETTE Lithium Heparin tubes for plasma biochemical analysis. Approximately 4 mL of blood was collected separately for serum biochemical analysis. Rat kidney, heart, liver, pancreas and spleen, were separately collected, weighed and kept in 10% formalin solution for histopathological observation. Plasma was separated from the blood via centrifugation at 1300 rpm for 10 min. Serum was separated by clot retraction. Biochemical parameters evaluated include plasma glucose, glycated haemoglobin, albumin, serum AST and ALT. Standard established methods were employed for the determination of the above-mentioned parameters [6].

#### 2.8.3. Oral Glucose Tolerance Test (OGTT)

An oral glucose load of 3 g kg^−1^ body weight was fed to the experimental rats. Blood glucose was measured at time intervals of 0, 30, 60, 90 and 120 min after being treated with glucose. Blood samples were obtained by pricking the tip of each rat’s tail with a 26G needle, and glucose levels were assessed with blood glucose test strips on an Accu-Chek Instant S glucometer (Roche Diabetes Care, Inc., Indianapolis, IN, USA) [6].

#### 2.8.4. Statistical Analysis

All the experiments were performed in triplicate, and the findings were represented as Mean SD. Antidiabetic data were further analysed using one-way ANOVA via GraphPad Prism 8.2.1.

## 3. Results and Discussion

### 3.1. Proximate Composition

Molecular identification of collected seaweed confirmed the species as *P. tetrastromatica*. Collected samples yielded 1.73% EtOH and 0.25% EtOAc crude extracts of the dry specimen. The proximate composition and gross calorific value of the specimens analysed were based on a dry weight basis (%) and presented in (Table 2). The moisture content of *P. tetrastromatica* was at 6.90 ± 0.09% DW. Moisture level assures the stability and quality of other chemical components in seaweeds as it prevents microorganisms from growing and allows longer storage times without loss of quality [11].

High ash content is an essential characteristic of seaweeds, and they contribute significant mineral elements (8–40%) required for human and animal nutrition [12,13]. A total 63.81 ± 0.26% ash composition was quantified in the current analysed *P. tetrastromatica*. According to Chan et al. (2017), the ash content of macroalgae is higher than that of the most common vegetables, due to the extraordinary ability of seaweed to accumulate minerals present in the water [14]. It is noteworthy that the ash content of seaweeds is generally higher than most common edible terrestrial vegetables. Sweet corn (2.6% DW), potatoes (10.4% DW), carrots (7.1% DW) and tomatoes (7.1% DW) because of their ability to accrue minerals present in the surrounding water bodies [14,15].

### 3.2. Protein and Amino Acid Composition

In general, the protein composition in red algae is recorded as the highest of the algae in the range between 14–47% DW. The protein level of the green algae is valued as intermediate in the range of 7–27% DW [16]. The brown algae contain the lowest percentage so far reported to be in the range of 5–19% DW) [4]. The protein content of *P. tetrastromatica* was quantified at 5.89 ± 0.70% DW, lower than the recorded range. Though low in protein content, seaweed is a crucial source of essential amino acids (EAA) required for the synthesis of protein, nutrient absorption and tissue repair mechanism. Among the common EAAs associated with seaweed are histidine, isoleucine, leucine, lysine, methionine, phenylalanine, threonine, tryptophan and valine. The amino acid composition of *P. tetrastromatica* is summarised in Table 2. The EAAs in *P. tetrastromatica* adds up to a total of 61.02 ± 221.92 mg g^−1^ corresponding to the crude protein value obtained with detection of leucine (36.5%), lysine (29.9%), threonine (13.1%), valine (11.5%), isoleucine (10.5%). Ingestion of EAA from food is extremely important for humans, due to the inability of the body to synthesise these amino acids.

The nonessential amino acids (NEAAs) profile is often rather consistent across the board, with approximately 20–32% of aspartic and glutamic acid from the total amino acid that is responsible for the typical taste and flavour of the seaweeds [4]. A total mean of 1017.86 ± 56.37 mg 100 g^−1^ EAAs was quantified in *P. tetrastromatica*. As highlighted by literature, from a total of 5083.77 ± 197.62 mg 100 g^−1^, glutamic and aspartic acids were the most abundant NEAAs, accounting for 11.1% and 40.5%, respectively. The sum of aspartic and glutamic acids was higher than data reported by other authors [17], who found values between (30.67–34.20%) in different brown seaweed species. The total protein composition in *P. tetrastromatica* is comparable to other terrestrial resources, such as cornmeal (41.3% DW), rice meal (40.9% DW), and soybean meal (40% DW) [18]. However, in this study, the average protein value was lower than other data recorded in other sources of brown algae [2,13,19].

### 3.3. Carbohydrate and Dietary Fibre

The carbohydrate concentration was calculated by subtracting the lipids, protein, ash, and moisture content from 100%. A total of 23.16 ± 0.44% DW was calculated in the analysed *P. tetrastromatica*. Carbohydrate, such as sugar, starch or fibre, is a major component in all seaweeds with the function of a photosynthetic reserve and osmoregulators, contributing as the energy source that prevents protein being used as energy and for fat metabolism [4,20]. Carbohydrate synthesis in edible seaweed is related to periods of maximum growth, increased photosynthetic activity and a reduction in protein content [21].

Seaweeds are a richer source of total dietary fibre containing between (29–62% of DW) when compared to most fruits, vegetables and whole food crops, i.e., barley, brown rice, flaxseed, and wheat germ [22]. However, they do not contribute much of the starchy carbohydrate to a diet that is obtained in brown rice, possibly leading to a negligible glycemic load [23]. Instead, high intakes of dietary fibre are consistently correlated to a reduced incidence of type 2 diabetes mellitus [9]. Values obtained for *P. tetrastromatica* was lower compared to literature with total dietary fibre quantified at 22.35 ± 6.69% DW [12]. The total dietary fibre content of several other macroalgae, such as *Ulva rigida*, *Gracilaria* sp., *Fucus vesiculosus* and *Saccharina latissima*, were in the range of 36.6 ± 1.5% to 45.0 ± 0.1% of DW [24]. Algae fibre differs chemically and physically from the fibre content of land-dwelling plants, and thus, induces different physiological effects [14]. As recommended by the World Health Organisation, the daily adequate intake (AI) of dietary fibre of Asians is between 25–38 g per day for adult women and men [25].

### 3.4. Total Lipid and Fatty acids (FA) Profile

Seaweeds are low-fat containing plants [12] that make edible seaweeds a low-calorie food source [19]. Being low in calories and rich in dietary fibre, unsaturated fatty acids and vitamins, seaweed act as a suitable alternative for managing diabetes [9]. The total lipid content of *P. tetrastromatica* was low, calculated at 0.24 ± 0.05%, and its calorie value was quantified as 10.51 ± 0.08 kcal kg^−1^.

With respect to fatty acids profile, total fatty acid content, was expressed as gram (g) FAME 100 g^−1^ total fat. The total FA in *P. tetrastromatica* was 1.87 ± 0.03 g 100 g^−1^ (Table 3). The saturated fatty acids (SFAs) detected were myristic acid (C14:0), palmitic acid (C16:0), stearic acid (C18:0) and arachidic acid (C20:0). The SFAs profile is comparable to most terrestrial plants more than that of aquatic species. With respect to monounsaturated fatty acid (MUFA) and polyunsaturated fatty acid (PUFA), only oleic acid (C18:1ω-9) and linoleic acid (C18:2n-6) was present, respectively. The MUFA detected was comparable to other brown seaweeds with oleic acids reported as the dominant MUFAs [17,24]. Studies show that diets rich in MUFAs is important because it helps to improve the fluidity of high-density lipoproteins, regulate blood cholesterol levels, as well modulate immune function [14]. High levels of MUFA were found to improve insulin sensitivity in healthy and glucose-intolerant candidates [9]. Similarly, the presence of ω-6 PUFA from plant sources has been known to trigger positive effects on insulin sensitivity and is associated with a lower risk of developing type-2 diabetes mellitus. Lorenzo et al. (2017) reported that PUFAs were the predominant fatty acids found in three Phaeophyta species, including *Fucus vesiculosus*, *Bifurcaria bifurcatain*, and *Ascophyllum nodosum* [17]. Contrarily only one PUFA species was detected, constituting of 0.1 ± 0.00 g 100 g^−1^. Overall, SFAs constituted 73.20%, MUFA at 21.44%, and PUFA was totalled at 5.36% of the total methylated fatty acid. The present study shows that *P. tetrastromatica* is devoid of any trans-fat-associated health risk. Based on the WHO recommendations, not more than 1% of our daily energy intake must come from trans-fatty acids [26].

### 3.5. Mineral Profile

Table 4 showed a detailed summary of the mineral composition of the studied seaweeds. The macro-elements were quantified at 366.97 mg 100 g^−1^ for *P. tetrastromatica*. Seaweeds are an important source of minerals and trace elements, reported to contain between 8–40% of the dry weight, due to their ability to absorb and accumulate these elements. A significant amount of calcium (Ca), quantified at 4388.3 mg 100 g^−1^, was detected for *P. tetrastromatica*. It is also noteworthy that the ratio of sodium/potassium (Na/K) in the algae was low, at 0.87, therefore, is advantageous to prevent hypertension [6]. Seaweeds with low ratios of Na/K are ideal for sodium chloride replacement. Iron (Fe) and manganese (Mn) composition were calculated to be 37.30 mg 100 g^−1^ DW and 326.34 mg 100 g^−1^ DW, respectively. The Fe content in this brown alga is higher than several terrestrial vegetables (2–4 mg 100 g^−1^), including legumes, cereal grains, nuts, and green leafy vegetables. As it is required for haemoglobin and myoglobin production, its deficiency is characterised by anaemia causing symptoms, such as fatigue and body weakness [27]. The composition of Mn in seaweed is associated with maintaining osmotic balance, ion regulation, and enzyme catalysis. However, high concentrations can be extremely toxic for consumption. Mn is a cofactor of several metalloenzymes and is associated with lipid, amino acid, and carbohydrate metabolism [28]. *Padina tetrastromatica* contained high concentrations of aluminium (530.64 mg 100 g^−1^ DW), arsenic (0.49 mg 100 g^−1^ DW) and lead (0.79 mg 100 g^−1^ DW).

The cell wall polysaccharides of seaweeds contain multiple functional groups, such as anionic carboxyl, sulfhydryl, sulphate, amino, and phosphate. These functional groups are accountable for their high complexation of metallic cation from their aquatic surroundings [29]. Among major macro elements, Ca remains the most abundant and accumulates in seaweeds at much higher levels than terrestrial food sources [23], not only to prevent bone-related diseases, but is necessary for intracellular functions and blood clotting hemostasis [27]. Chromium (Cr), detected at 0.65 mg 100 g^−1^, is a key element in carbohydrates metabolisms and helps to boosts insulin activity [30]. Cobalt (Co) is essential for the synthesis of vitamin B12, which in turn, is a key coenzyme in cytosolic transmethylation of homocysteine and propionate metabolism. Molybdenum (Mo) is crucial to the activity of certain enzymes, such as xanthine oxidoreductase and sulphite oxidase that catalyse redox reactions, whereas selenium (Se) is mainly present as part of selenoproteins, which have a variety of functions, including T-cell immunity, antioxidant effects, thyroid hormone metabolism, and skeletal and cardiac muscle metabolism [31]. Copper (Cu) and zinc (Zn) are vital components of numerous enzymes, including those involved in energy metabolism, neurotransmitter synthesis, and collagen/elastin cross-linking [28]. Differences in the treatment of materials, analytical procedures and sample location, makes it difficult to adequately compare data of previous studies [32]. Overall, the variety of mineral and trace elements in seaweed in general and *P. tetrastromatica* in specific makes them one of the best approaches to address nutritional deficiencies.

### 3.6. Nonnutritive Components and Biological Activities

Many synthetic antioxidants, including butylated hydroxytoluene, butylated hydroxyanisole, propyl gallate, and tert-butylhydroxyquinone have been widely used in food and pharmaceutical industry products. However, due to their potential health hazards, their application as food additives is under regulation in several countries [33]. Interestingly, natural antioxidants, including polyphenols and flavonoids, have demonstrated a positive effect on human health [13]. Moreover, the antioxidant activity measured by an individual assay reveals the chemical reactivity under the specific conditions employed in that assay. Therefore, assessing the seaweed extract can provide an overall idea of their antioxidant activity.

The total phenolic content of the dried *P. tetrastromatica* was quantified at 61.20 ± 3.37 mg GAE g^−1^. The total flavonoid content was determined as 382.06 ± 2.72 mg QE g^−1^.The antioxidant ability for reducing Fe (III) to Fe (II) of *P. tetrastromatica* was at 30.39 ± 3.63 µg AAE g^−1^. The presence of possible reductants in the extract reduces the Fe^3+^/ferricyanide complex to its Fe^2+^ form, in a redox-linked colourimetric reaction by electron transfer, which can be monitored by measuring the formation of Perl’s Prussian blue at 700 nm [34]. The reducing capacity assay exhibits the reducing capacity of the seaweed extract, which indicates the presence of reductones, that are terminators of free radical’s chain reactions [15]. The total antioxidant activity of *P. tetrastromatica* was 114.55 ± 2.91 mg GAE g^−1^ DW. The high radical scavenging ability exhibited by the extract was connected to the presence of aliphatic alcohol, diterpenes, hexadecanoic acid, cholest-5-en-3-ol, sterols isolated from studied seaweeds [35]. The H_2_O_2_ scavenging activity of the *P. tetrastromatica* extracts was quantified at 76.41 ± 0.01%. The H_2_O_2_ scavenging activity assay is important to understand the scavenging potential of the seaweed extract against H_2_O_2_, a nonradical compound capable of permeating biological membranes and causing toxic damage to the cells [36].

### 3.7. In Vivo Antidiabetic Study

The screening of extracts was conducted in animal models because the processes and mechanisms leading to diabetes and its complications involve more than one organ. The use of in vitro studies that targets a specific cell line or a specific organ seldom gets translated into meaningful *in vivo* outcomes, especially in diabetes mellitus. In vivo studies tailor research questions towards individualised genetic and biochemical contributors and their effect on the pathogenesis of the disease [37].

#### 3.7.1. Effect of *P. tetrastromatica* Extracts on the Body Weight of Rats

**Figure 1:**
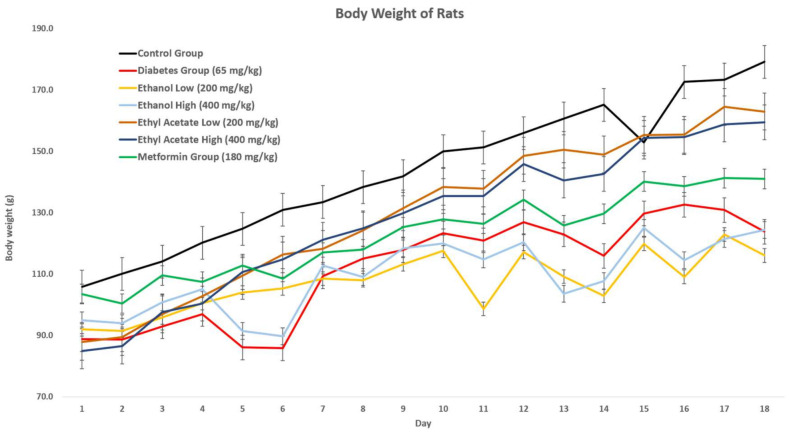
Effect of *P. tetrastromatica* extracts on the body weight of diabetes-induced rats.

After 72 h introducing STZ, the rats began to show a decrease in their body weight and increase in food and water intake. The body weight lowering effect was observed in all animals that have been induced with diabetes. This is due to muscle wasting in a diabetic state [38]. Body weights of all diabetic induced animals were monitored and documented for up to 18 days, and the results are shown in Figure 1. Throughout this study, the body weight of animals in the control group increased constantly, while the body weight of animals in the STZ-induced group experienced weight loss, due to STZ-toxicity. Based on the graph below, the treatment of *P. tetrastromatica* extracts (EtOH and EtOAc) and metformin successfully prevented weight loss in diabetic rats as no significant decrease in body weight was observed in the treatment groups. Comparatively, the diabetic rats treated with both high (400 mg kg^−1^) and low (200 mg kg^−1^) dose of EtOH extract showed a significant reduction in their body weights, similar to the diabetic control group. Throughout this study, the overall body weight of animals showed an increasing trend.

As observed in the experiment, the general pattern of body weight was positive. However, with the induction of diabetes, the increase in body weight was slightly interrupted, causing a slight decrease in change. Weight loss is a commonly observed symptom in both types of diabetes where the decreased insulin production prevents body cells from breaking down glucose to obtain energy [38]. The body compensates for the ineffective utilisation of glucose by breaking down the fats and muscles, leading to an overall reduction in body weight. Instead of burning their own fats and muscle, increasing food intake do help them in obtaining more energy. According to Ponnanikajamideen et al. (2017), treatment with *P. tetrastromatica* extracts had proven to show a significant increase in body weight and a significant decrease in food intake among the diabetic rats [39]. However, the normal control group showed the largest increment representing the batch of healthy rats. On the contrary, the body weight of STZ-induced rats displayed an erratic trend of weight loss and little gain throughout the 18-day observation period, which was caused by STZ-toxicity. In our study, the varying doses of ethanolic extracts somehow displayed a similar erratic trend as the diabetic induced rats, with no significant effect in preventing weight loss. The metformin and varying doses of *P. tetrastromatica* EtOAc extracts treated group displayed a consistently upward increasing trend in body weight, indicating prevention of weight loss in diabetic rats with the administration of test samples. A comparison between the extract doses observed an almost identical body weight retention pattern via administration of EtOAc extracts regardless of the dose administered.

#### 3.7.2. Effect of *P. tetrastromatica* Extracts on Food Intake

**Figure 2:**
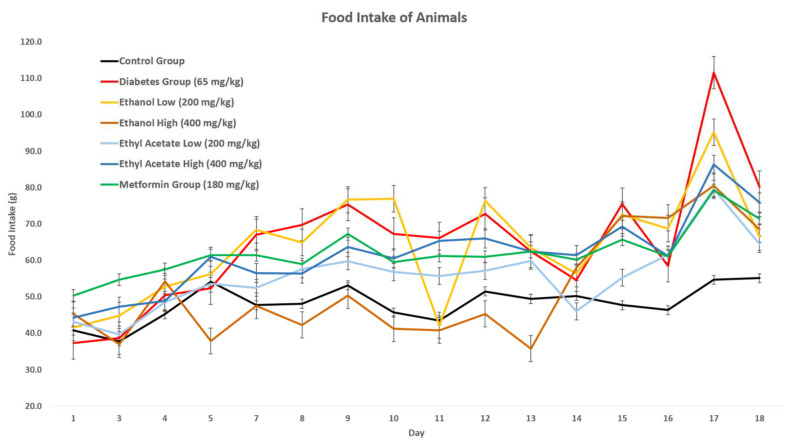
Effect of food intake on *P. tetrastromatica* extract-treated animals.

Food intake in all animals was recorded daily throughout the study. Based on Figure 2, a significant increase in food intake, approximately up to 50 g, was observed in the animals of the STZ-induced group, compared to the control, which was consistently in the range of 40–50 g. When *P. tetrastromatica* extract treatment was administered, their food intakes were significantly reduced as compared to the STZ-induced group and their body weight started to increase constantly. This observation has come in agreement with the study conducted by Granneman et al. (1984), which proposed that hyperphagia is common after administration of STZ, which induces diabetes [40]. Diabetic rats increase their food intake, due to enhancement of gastric emptying and rapid digestion of intestinal carbohydrates.

Diabetic organisms compensate for the ineffective utilisation of glucose by breaking down the fats and muscles, leading to an overall reduction in body weight [41]. The increasing food intake contributes to obtaining more energy. According to Ponnanikajamideen et al. (2017), *P. tetrastromatica* extracts had proven to show a significant increase in body weight and a significant decrease in food intake among diabetic rats [39]. Upon initiating treatments of EtOAc extracts of *P. tetrastromatica* and metformin, a significant reduction in food consumption was observed as the animals consumed a lesser amount of food, compared to the animals in the STZ-induced group. In contrast, the treatment of EtOH *P. tetrastromatica* extract showed no significant reduction in food consumption.

Both the seaweed extracts and commercial drug-maintained food intake were lower than the untreated STZ-induced rats. Initially, both the EtOH and EtOAc extract-treated animals recorded food intake measurements close to normal rats, but had a spike from the 13th day of the experiment. The relatively low food intake by EtOAc administered rats compared to the untreated rats could also be due to the possible distasteful nature of extracts. Since carbohydrate is generally regarded to be easily absorbed, metabolised and stored with less bioenergetic efficiency than dietary fat, ingestion of EtOH extract could contribute to the reduced appetite and urge to eat [42]. Through observation in food intake shows a moderate consumption upon treatment of extract, overlaying data from weight change indicates that the *P. tetrastromatica* EtOAc extract has potential as an antidiabetic agent.

#### 3.7.3. Effect of *P. tetrastromatica* Extracts on Plasma Glucose and Glycated Haemoglobin Levels

The plasma glucose level and percentage of glycated haemoglobin in the experimental rats were quantified after 18 days of treatment with *P. tetrastromatica* extracts. Based on the level obtained, the STZ-induced group showed significantly (*p* < 0.05) high plasma glucose levels caused by diabetes induction as compared to normal rats. However, after administration of varying doses of *P. tetrastromatica* EtOAc extracts, a significant reduction in plasma glucose level was observed, indicating the potential to reverse the elevated plasma glucose levels in diabetic rats. A similar pattern was observed in the percentage of glycated haemoglobin, indicating the potential antihyperglycaemic effect of the *P. tetrastromatica* EtOAc extract. The reduction in plasma glucose level by the EtOH extract-treated group was not significant.

**Figure 3:**
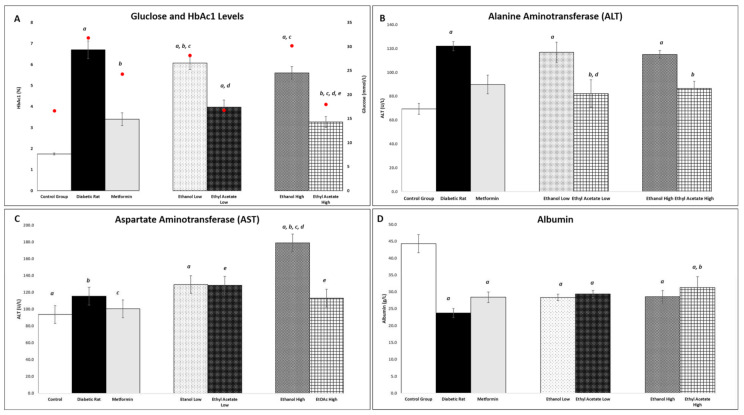
Effect of *P. tetrastromatica* extracts on plasma glucose and HbA1C levels (**red dot**) (**A**), alanine aminotransferase (ALT) (**B**), aspartate aminotransferase (AST) (**C**) and albumin (**D**) after 18-day treatment. (Note: For each group, high represents 400 mg kg^−1^ dose, and low represents 200 mg kg^−1^ dose of the respective extract. Significant differences (*p* < 0.05) were observed between groups and is indicated with the following letters; *a*—against Control group, *b*—against Diabetic group, *c*—against metformin administered group, *d*—against Ethanol low administered group, *e*—against Ethanol high administered group.

*Padina tetrastromatica* extract can reduce blood glucose levels, which can be associated by the seaweeds’ role in increasing insulin secretion and enhancing glucose uptake by adipose and skeletal muscle tissue for the utilisation of energy expenditure [43]. *Padina tetrastromatica* has been reported to display the ability to scavenge free radicals and to inhibit lipid peroxidation that prevents STZ-induced oxidative stress and protects β-cells [39]. This promotes increased insulin secretion and decreased plasma glucose levels, an essential trigger for the liver to revert its normal homeostasis during diabetes. The consumption of dietary MUFA is also known to improve insulin sensitivity in rats [9]. Seaweeds are well-known antioxidant agents with the presence of compounds, such as pigments and polyphenols comprising of flavonoids, phenolics and tannins. These compounds are the causative agents for their pharmacological effects, such as the antihyperglycemic properties exhibited in these experiments [44]. Mohan et al. (2014) had mentioned that when *P. tetrastromatica* extracts were given to STZ-induced rats, their elevated fasting glucose were reversed to normal levels [45]. The reduction in these plasma glucose levels has been strongly related to the ability of *P. tetrastromatica* extract to trigger proinsulin synthesis and also insulin release [39]. Thus, *P. tetrastromatica* was proven to show an antihyperglycaemic effect in reducing the plasma glucose levels in diabetic rats. Figure 3 shows the levels of plasma glucose level and percentage of glycated haemoglobin in the experimental rat samples.

#### 3.7.4. Effect of *P. tetrastromatica* Extract on Plasma ALT and AST Levels

According to Mohamed et al. (2016), the liver is one of the primary organs susceptible to the effects of hyperglycaemia-induced oxidative stress caused by diabetes induction which can lead to liver tissue injury and leakage of hepatic enzymes, such as ALT [46]. In this study, a significant reduction in ALT level was observed in the *P. tetrastromatica* EtOAc extract-treated group compared to the ethanol extract. This showed that the *P. tetrastromatica* EtOAc extract can reverse the elevated ALT levels in diabetic animals and possess a protective effect on liver cells. The liver is one of the primary organs susceptible to the effects of hyperglycaemia-induced oxidative stress, which can lead to liver tissue injury [46]. ALT is a good serum biomarker specifically used to indicate liver damage, while AST can be used as a biomarker in detecting liver injury, coronary heart disease or muscle injury [47]. The elevated levels of ALT and AST are common in diabetics, which both are commonly associated with insulin resistance or metabolic syndrome [48]. Similarly, the AST level was monitored, and the STZ-induced group exhibited higher levels than the control group, but there were no significant differences between them. Upon administration of *P. tetrastromatica* extract, no significant changes were observed in both 200 mg kg^−1^ and 400 mg kg^−1^ groups as compared to the STZ-induced group. This is because AST is normally found in a variety of tissues the including the liver, heart, muscle, kidney and brain. Therefore, AST is not a highly specific indicator of liver injury [47]. The ALT and AST levels of the diabetic rats are shown in Figure 3B,C.

#### 3.7.5. Effect of *P. tetrastromatica* Extracts on Plasma Albumin Levels

Nephropathy is one of the major complications faced by diabetics where their kidney functions are impaired, leading to glomerular hyperfiltration and increased albumin secretion [49]. The induction of diabetes has a damaging effect on the levels of albumin. According to Lim et al. (2014), microalbuminuria is an early marker present in both types of diabetics, in which the glomerular permeability of the kidney was increased, and the albumin reabsorption at the proximal convoluted tubule was decreased, leading to increased albumin secretion, which eventually developed into overt nephropathy [49]. In this case, the STZ-induced group went through a 50% reduction in albumin level, recording the lowest amongst all experimental rats, which shows the presence of microalbuminuria where the kidneys begin to remove excessive albumin from the blood. Administration of metformin, a commercial antidiabetic drug, managed to restore 19% of the substance, recording a concentration of 28.4 g L^−1^. We observed that the introduction of EtOH and EtOAc extracts restored albumin levels within the range of metformin. Among the *P. tetrastromatica* extracts, only 400 mg kg^−1^ of EtOAc extract-treated group showed significantly increased albumin levels, which substantiates the presence of antihyperglycaemic potential in *P. tetrastromatica* as it also showed effect in controlling the development of nephropathy in diabetic conditions. Interestingly, the *P. tetrastromatica* EtOAc extract at the dose of 400 mg kg^−1^ showed the greatest increase in albumin levels at 31.9% compared to the metformin in the STZ-induced group, making it a potent alternative in restoring the normal functions of and possess a protective effect on the kidney. Figure 3D shows the concentration of albumin levels evaluated in this study.

#### 3.7.6. Histopathological Analysis

**Figure 4:**
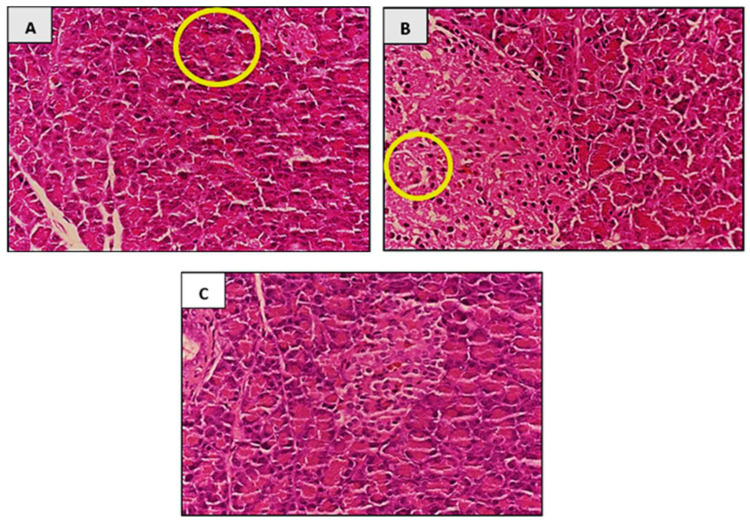
Histopathological analysis of pancreas after 18 days of treatment (EtOAc extract 400 mg kg^−1^ dose); (**A**) Presence of islet atrophy in STZ-induced group. (**B**) Presence of insulitis in the STZ-induced group. (**C**) No presence of cellular atrophy and insulitis in the group treated with *P. tetrastromatica* extract.

After 18 days of treatment, histopathological analysis was done on all the organs collected. Figure 4A–C shows histopathological analysis of pancreas in experimental rats after 18 days of *P. tetrastromatica* EtOAc extract treatment. No significant changes were observed in the histopathological morphology of all organs collected from the control group and both groups treated with *P. tetrastromatica* extracts. However, the presence of insulitis and atrophy were observed only in the pancreas of the STZ-induced group.

It was evident that only the pancreas of the STZ-induced group showed histopathological morphology changes where insulitis and cellular atrophy were both observed in the pancreas. This might have been due to the cell-mediated autoimmune reaction directed against the pancreatic β-cells [50]. Besides the pancreas, no changes were observed in other organs. This study was performed for 18 days and this period is too short of showing visible changes in their histopathological morphology. For *P. tetrastromatica* extract-treated group, no significant changes as compared to control was observed in the histopathological morphology of the ethanol extract (high and low doses) and low dose ethyl acetate treated rats. However, we observed that the high dose EtOAc treated rats had demonstrated preventive effects in liver damage caused by diabetes, as shown in Figure 4C, compared to the affected cell in Figure 4B. This implies that *P. tetrastromatica* EtOAc extract restores pancreas histology by alleviating oxidative stress induced by STZ and potentially increases the size of the islets by its ability to regenerate β-cells.

All evaluations in the *in vivo* experiments indicate a positive effect of the extract against diabetes. In a comparison between other brown algae, the antidiabetic potential of two edible seaweed *Sargassum polycystum* and *Sargassum wightii*, also showed possible mechanisms of antidiabetic action in vitro and *in vivo*. The study by Unnikrishnan et al. (2015) confirms that the extracts of *S. polycystum* and *S. wightii* have significant effects in inhibiting major carbohydrate-hydrolysing enzymes, such as α-amylase and α-glucosidase, and inhibiting incretin-degrading enzymes, such as dipeptidyl peptidase IV, which can delay carbohydrate digestion and glucose absorption by preventing postprandial hyperglycaemia [51]. The study by Lee et al. (2008) affirms that bioactive compounds from edible seaweeds have significant roles in the modulation of glucose-induced oxidative stress and inhibition of starch-digestive enzymes [52].

### 3.8. Presence of Bioactive Components in P. tetrastromatica

**Figure 5:**
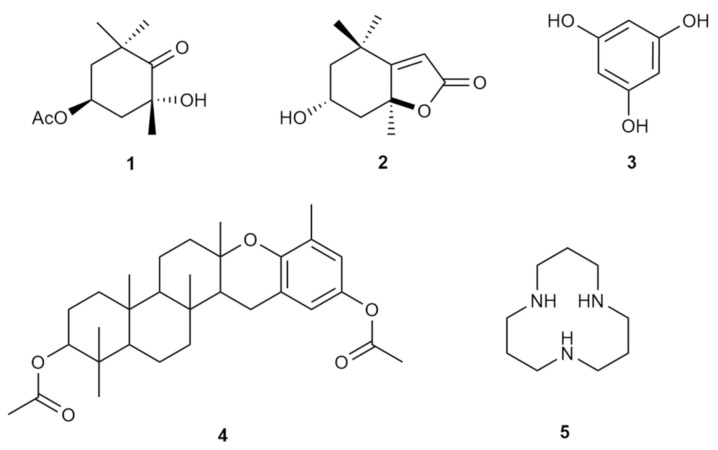
Secondary metabolites from *P. tetrastromatica*.

To date, diabetes mellitus is managed using chemical or biochemical agents, such as insulin, biguanides, sulfonylureas, thiazolidinediones and α-glucosidase inhibitors. Unfortunately, the use of these therapeutic agents comes with the risk of undesirable adverse events on the course of recovery, among which are weight gain, sour stomach, belching, nausea, vomiting, indigestion, and diarrhoea. Several natural bioactive components have been reported to date that have the potential to be used in the treatment and management of diabetes mellitus. Most of these bioactive compounds are safer and have minimal adverse effects. For instance, metformin, which was used in this investigation, is derived from *Galega officinalis*. French lilac is typical for treating diabetes, which has been used as a first-line drug for type 2 diabetes mellitus for 60 years [53]. However, this drug can result in contradictions in renal insufficiency, while some clinical therapeutic drugs have been reported to elicit, severe gastrointestinal problems. Therefore, new sources of natural alternatives are necessary to facilitate the cure of this rapidly growing condition. As such, seaweeds are globally gaining recognition as an important functional food, due to their rich nutritional properties and source of bioactive compounds is the way forward for exploration for therapeutics [43]. Antidiabetic properties in seaweed have been closely linked to the diverse chemical constituents natural synthesised, such as alkaloids, glycosides, polyphenols, carotenoids, terpenoids, flavonoids, anthocyanins, tocopherols, peptidoglycans, steroids, saponins, xanthones, and polysaccharides [54]. The active constituents in the genus *Padina* have been reported to be bromophenols, halogenated terpenoids and sulphated polysaccharides [7,39,55,56]. Halogenated compounds are known to promote bioactivity in secondary metabolites, and sulphated polysaccharides have been strongly associated with antidiabetic properties, as exhibited in Ponnanikajamideen’s research. Among the metabolites isolated from *P. tetrastromatica* are (2R,4S-4-acetoxy-2-hydroxy-2,6,6-trimethylcyclohexanone (1), loliolide (2) [55], phloroglucinol (3), polcyclic mero diterpenoid (4), 1,5,9-triaza-cyclododecane (5) [56]. The compounds structures are shown in Figure 5. Regardless, despite the availability of existing bioactive natural compounds for the management of diabetes mellitus, further mechanisms of action, bioavailability, and safety remain to be deeply explored.

## 4. Conclusions

Diabetes mellitus is a metabolic disorder associated with complications, such as liver damage, impaired kidney function and coronary heart diseases that affect the quality of life. Hence, management of diabetes is essential in assuring ourselves a healthy life. *P. tetrastromatica* used in this study are renewable resources that are economically valued and easily accessible. The high dose ethyl acetate extract of *P. tetrastromatica* had successfully initiated the restoration of the liver and kidney from diabetes-induced oxidative stress in the STZ-induced rats indicating potential antidiabetic character, antioxidant and antihyperlipidemic properties in the STZ-induced diabetic rats. The biochemical studies were supported by histopathological studies. With this possibility, further studies on biologically active secondary metabolites of *P. tetrastromatica* can be done to identify active components in the plant to become an alternative treatment approach for diabetes patients in the future. Marine alga is an uprising source of functional food with various biological and nutritional properties essential for humans. Thus, this natural resource must be sustainably managed and cultivated for future stock and application in mariculture, pharmaceuticals and nutraceuticals worldwide.

## Figures and Tables

**Table 1:** Grouping of the animals used to determine the antihyperglycaemic potential of *P. tetrastromatica* extracts.

Group	Group Description
Group I	Normal control group
Group II	Diabetes-induced group (65 mg kg^−1^)
Group III	Ethanol extract-treated group (200 mg kg^−1^)
Group IV	Ethanol extract-treated group (400 mg kg^−1^)
Group V	Ethyl acetate extract-treated group (200 mg kg^−1^)
Group VI	Ethyl acetate extract-treated group (400 mg kg^−1^)
Group VII	Metformin treated group (180 mg kg^−1^)

**Table 2:** Amino acid composition in the brown algae *P. tetrastromatica* (*n* = 3).

**Essential Amino Acids (EAAs)**	**Composition (mg g^−1^)**
Threonine	1.319 ± 0.009
Valine	1.155 ± 0.003
Isoleucine	1.049 ± 0.106
Leucine	3.659 ± 0.187
Lysine	2.997 ± 0.102
Sum TEAAs	10.179 ± 0.06
**Nonessential Amino acids (NEAAs)**	**Composition (mg g^−1^)**
Aspartic acids	24.704 ± 0.218
Glutamic acid	6.829 ± 0.086
Serine	0.47 ± 0.001
Glutamine	10.307 ± 0.086
Glycine	4973.57 ± 0.129
Alanine	0.150 ± 0.003
Cysteine	0.247 ± 0.003
Tyrosine	2.307 ± 0.02
Arginine	1.233 ± 0.06
Sum TNEAAs	50.838 ± 0.19
EAAs/TAAs (%)	16.68 ± 0.05
NEAAs/TAAs (%)	83.32 ± 0.05
EAAs/NEAAs (%)	20.02 ± 0.07
Chemical score	0.478

Mean ± standard deviation of three replicates; DW, dry weight; TEAA, total essential amino acids; AAs, essential amino acids; The, threonine; Val, valine; Ile, isoleucine; Leu, leucine; Lys, lysine; NEAAs, nonessential amino acids; Asp, aspartic acids; Glu, glutamic acid; Ser, serine; Glu, glutamine; Gly, glycine; Ala, alanine; Cys, cysteine; Tyr, Tyrosine; Arg, Arginine; TAA, total amino acid; TNEAA, total nonessential amino acids.

**Table 3:** Fatty Acid composition of analysed *P. tetrastromatica*.

Fatty Acid	Content (g 100 g Oil^−1^)
**SFAs**	
Myristic acid (C14:0))	0.1 ± 0.00
Palmitic acid (C16:0)	0.83 ± 0.06
Stearic acid (C18:0)	0.3 ± 0.00
Arachidic acid (C20:0)	0.10 ± 0.00
Total (%)	1.37 ± 0.07 (73.20 ± 2.88)
**MUFA**	
Oleic acid(C18:1ω9)	0.40 ± 0.10
Total (%)	0.4 ± 0.05 (21.44 ± 2.83)
**PUFA**	
Linoleic acid (C18:2 ω6) cis	0.10 ± 0.00
Total	0.1 ± 0.00 (5.36 ± 0.1)
Total FA	1.87 ± 0.03

DW, dry weight; PUFA, polyunsaturated fatty acids; MYFA, monounsaturated fatty acids; SFA, saturated fatty acids.

**Table 4:** Mineral composition in *P. tetrastromatica* sample (*n* = 3).

Minerals	Content (mg 100 g^−1^)
**Macro Metal**	
Calcium	4388.3
Magnesium	3.47
Potassium	171.3
Sodium	148.2
Na/K	0.87
**Trace Mineral**	
Copper	0.60
Iron	37.3
Manganese	326.34
Molybdenum	0.53
Selenium	0.068
Zinc	1.34
Chromium	0.65
Cobalt	0.14
**Heavy metal/TWIs**	
Total Arsenic	0.49
Cadmium	0.01
Aluminium	530.64
Lead	0.79

Triplicate measurements of each sample with RSD is less than 10% Ca (calcium); Mg, magnesium; K, potassium; Na, sodium; Cu, copper; Fe, iron; Mg, manganese; Mo, molybdenum; Se, selenium; Zn, zinc; Cr, chromium; Co, cobalt; As, arsenic; Cd, cadmium; Al, aluminium; Pb, lead.

## Data Availability

Additional data can be obtained from authors.

## Abbreviations

WWF	World Wildlife Fund
SPS	Sulphated polysaccharide
WHO	World Health Organisation
STZ	Streptozotocin
DNA	Deoxyribonucleic Acid
PCR	Polymerase chain reaction
AOAC	Association of Official Analytical Chemists
*v*/*v*	volume/volume
h	Hour
DW	Dry weight
Hex	Hexane
EtOAc	Ethyl Acetate
GC	Gas chromatography
FAME	Fatty acid methyl ester
FA	Fatty acid
HCl	Hydrochloric acid
HPLC	High-Performance Liquid Chromatography
HNO_3_	Nitric acid
H_2_O_2_	Hydrogen peroxide
ICP-MS	Inductively coupled plasma mass spectrometry
TPC	Total Phenolic Content
TFC	Total Flavonoid Content
FC	Folin–Ciocalteau
GAE	Gallic acid equivalent
QE	Quercetin equivalent
TAA	Total Antioxidant Activity
AAE	Ascorbic acid equivalent
OGTT	Oral Glucose Tolerance Test
AST	Aspartate Transaminase
ALT	Alanine Transaminase
SD	Sprague Dawley
ACUC	Animal Care and Use Committee
EAA	Essential amino acid
NEAA	Nonessential amino aid
TNEAA	Total Nonessential amino aid
TAA	Total Amino acid
PUFA	Polyunsaturated fatty acids
MUFA	Monounsaturated fatty acids
SFA	Saturated fatty acids
